# Exercise induced immune regulation and drug efficacy in rhinitis nasopharyngeal carcinoma implications for tumor microenvironment single cell immune signal transduction

**DOI:** 10.3389/fimmu.2025.1673383

**Published:** 2025-10-13

**Authors:** Guanwen He, Weijing Bao, Jiansheng Yang, Xiuqin Guo, Wenxian Lu, Xiuhui Ji, Shang Gao, Rifu Wei, Yisheng Chen

**Affiliations:** ^1^ Ningde Clinical Medical College of Fujian Medical University, Ningde, China; ^2^ Department of Otolaryngology, Ningde Municipal Hospital of Ningde Normal University, Ningde, China; ^3^ Department of Otolaryngology, Pingnan County General Hospital of Ningde Municipal Hospital Medical Group, Ningde, China; ^4^ Fujian Key Laboratory of Toxicant and Drug Toxicology, Medical College, Ningde Normal University, Ningde, China; ^5^ Department of Pediatrics, School of Pediatrics, Nanjing Medical University, Nanjing, China; ^6^ Department of Otorhinolaryngology, Shanghai General Hospital, Shanghai, China; ^7^ Department of Interventional Vascular Surgery, Ningde Municipal Hospital of Ningde Normal University, Ningde, China

**Keywords:** exercise, tumor microenvironment, immune signal transduction, single cell, immunotherapy, inflammation, nasopharyngeal carcinoma, allergic rhinitis

## Abstract

Emerging evidence reveals that exercise modulates immune signaling to enhance the efficacy of immunotherapy in diseases like allergic rhinitis (AR) and nasopharyngeal carcinoma (NPC). By influencing immune cell trafficking, reprogramming inflammatory pathways within the tumor microenvironment (TME), and altering drug pharmacokinetics, exercise improves immune responses and therapeutic outcomes. Exercise enhances immune cell activation and infiltration into tumors, modulates checkpoint and cytokine signaling cascades, and mitigates treatment-related side effects, thereby improving patient compliance. Recent advancements in single-cell technologies, such as single-cell RNA sequencing and spatial omics, provide unprecedented insights into immune cell heterogeneity and signal transduction dynamics in the TME, uncovering new targets for exercise-modulated therapies. This review explores the synergistic effects of combining exercise with immune-based therapies, particularly in cancer treatment, highlighting the role of exercise in reshaping TME inflammation, overcoming immune evasion, and enhancing immune-mediated drug bioavailability. Personalized exercise regimens, tailored to individual patient profiles, are critical for optimizing therapeutic responses. Integrating exercise with immunotherapy, guided by single-cell and systems-level analyses, may provide a transformative approach for improving the clinical outcomes of AR and NPC patients, paving the way for more effective, individualized cancer treatments.

## 1 Synergistic effects of exercise on drug efficacy and immunoregulation

### 1.1 Impact of exercise on drug metabolism

Exercise enhances drug metabolism by improving cardiac output and blood flow, facilitating tissue distribution and absorption (1, 2). This benefits targeted therapies, as shown with potassium losartan, where exercise-induced circulation improved dissolution and nasal delivery (3). Increased vascular permeability within the tumor microenvironment (TME) further optimizes intratumoral drug diffusion (4), aiding treatments for rhinitis and nasopharyngeal carcinoma (NPC) (5, 6).

Exercise also modulates the immune microenvironment, enhancing drug bioavailability and efficacy, as seen with ebastine in allergic rhinitis (7–10). By boosting T cell, B cell, and macrophage activity (11) and improving immune surveillance (12, 13), exercise supports therapeutic outcomes. Innovations such as transferosome oral films greatly increase ebastine absorption (14, 15), and when combined with exercise-induced immune activation, they strengthen targeted drug delivery for immune-related diseases, they strengthen spatiotemporal drug delivery and immune modulation at the single-cell level in immune-related diseases (16).

### 1.2 Immunoregulatory effects: the role of exercise in local immunity

Exercise boosts immune responses by enhancing immune cell function and migration, thereby remodeling the local TME in rhinitis and NPC (11, 17). In the nasal cavity, it improves circulation, aiding immune cell aggregation, allergen response, and drug absorption (18). For cancer, it enhances chemo- and immunotherapy efficacy inflammation regulation and single-cell immune signaling in the TME (19). Centipeda minima (CM) shows anti-inflammatory and antitumor effects, potentiated by exercise through better hemodynamics and immune activation (20, 21). In NPC, this synergy reduces immune suppression and improves tumor targeting (22). Exercise also reprograms immune tolerance in allergic rhinitis and NPC by boosting immune activity, antigen recognition, and pro-inflammatory cytokine release (23–25), thereby fostering a responsive immunotherapeutic microenvironment that can be mapped at single-cell resolution (26, 27).

### 1.3 Mechanisms of exercise-enhanced drug therapy

Exercise regulates inflammation, improving drug efficacy by increasing anti-inflammatory IL-10 and reducing TNF-α, IL-1β (28). creating a favorable immune milieu for therapy. Reduced inflammation optimizes immune function, drug permeability, and targeting (26). In rhinitis and NPC, exercise alleviates local inflammation, enhances immune cell migration/activation, and supports treatment (29, 30). Combined with immune checkpoint inhibitors like atezolizumab, it boosts tumor immune activity, improving efficacy (31). Mechanistically, exercise may modulate checkpoint pathways and antigen-presenting cell signaling cascades within the TME, thereby refining immune tolerance and mitigating immunotherapy side effects (19).

### 1.4 Exercise intensity, immune modulation, and drug efficacy

Exercise intensity modulates both immune function and pharmacological responses. Moderate-intensity exercise (MIE) enhances immune surveillance, attenuates inflammation, and facilitates drug absorption and distribution through increased natural killer (NK) cell activity, improved antigen presentation, and favorable cytokine shifts (11, 32). In allergic rhinitis (AR), MIE has been shown to improve intranasal drug penetration, while in nasopharyngeal carcinoma (NPC), it enhances the efficacy of immunotherapy. In contrast, high-intensity exercise (HIE) can induce transient immunosuppression, often referred to as the “open window” phenomenon (33). This state is characterized by reduced NK cell activity, decreased salivary immunoglobulin A (IgA), and elevated stress hormone levels, all of which may impair drug efficacy. From a pharmacokinetic perspective, MIE helps sustain therapeutic plasma concentrations, whereas HIE may accelerate drug clearance and consequently lower tissue drug exposure (34).Thus, exercise intensity critically dictates the balance between pro- and anti-inflammatory signaling within the TME, shaping both immune responses and drug effectiveness.

### 1.5 Impact of exercise on immune cell trafficking and drug resistance in NPC

Exercise modulates immune cell trafficking and overcomes immune resistance in NPC’s TME by enhancing blood flow and tissue perfusion, facilitating CD8^+^ T cell and NK cell infiltration (35). It can reduce PD-L1 expression on tumor and immune cells, improving T cell function and immune surveillance, while increasing IL-6 and TNF-α to activate effector T cells. Exercise also normalizes tumor vasculature, improves oxygenation, and reduces hypoxia, enhancing drug delivery and limiting Treg/MDSC accumulation (36). At single-cell resolution, these changes highlight how exercise remodels the inflammatory and immune signal transduction landscape of the TME, strengthening immune-mediated tumor destruction and improving therapeutic responses (37).

## 2 Exercise in rhinitis and nasopharyngeal carcinoma: modulating immunity and enhancing drug efficacy

### 2.1 Exercise and immune regulation in allergic rhinitis

**Figure 1:**
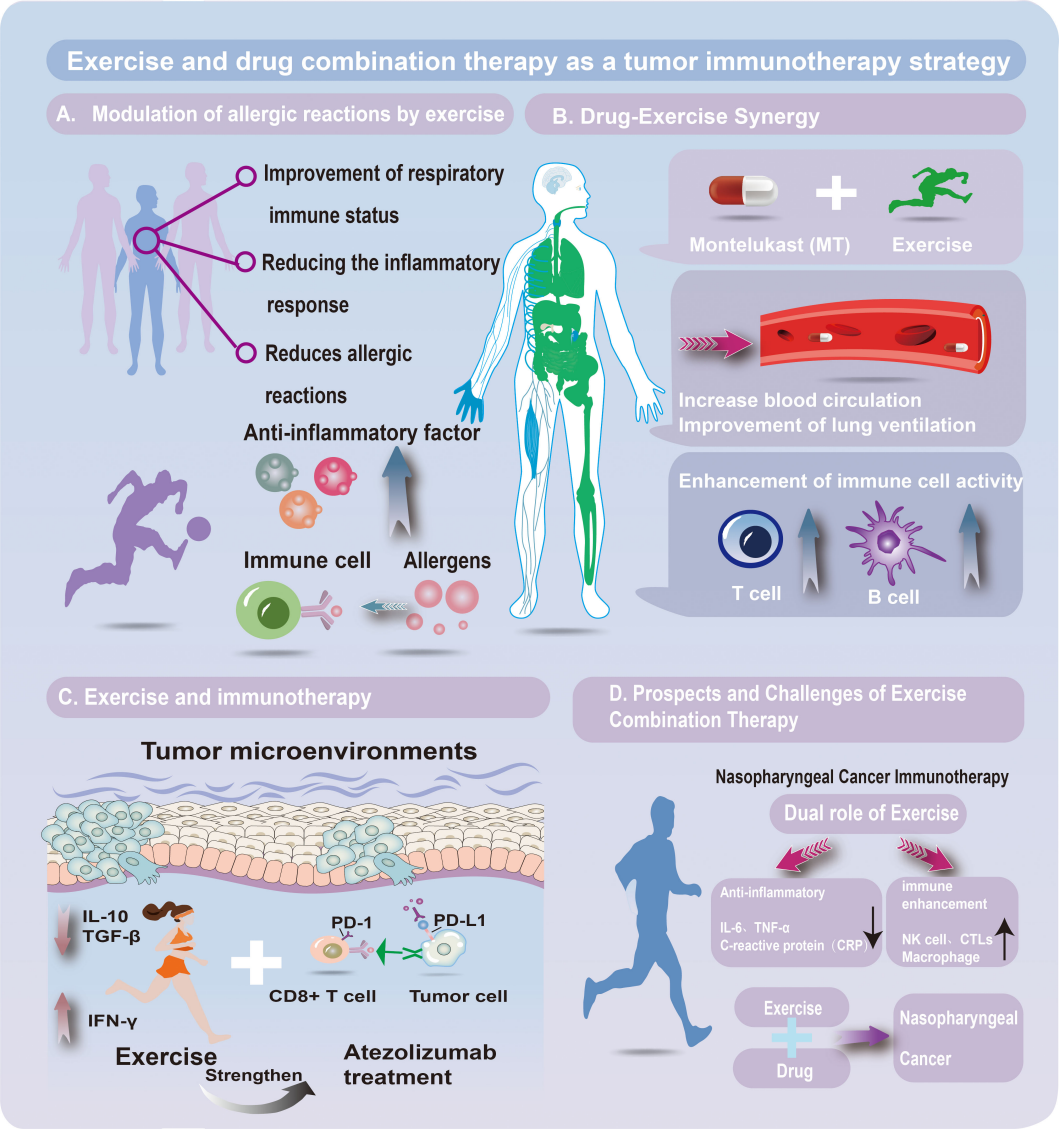
EExercise–drug combination therapy in tumor immunotherapy. **(A)** Exercise and allergy modulation. Exercise enhances respiratory immunity, lowers inflammation, and reduces allergic reactions via anti-inflammatory mediators and allergen-responsive immune cells. **(B)** Drug–exercise synergy. Combining montelukast (a leukotriene receptor antagonist) with exercise improves circulation and ventilation, boosting T- and B-cell activity and strengthening immune defense against tumors and inflammation. **(C)** Exercise and immunotherapy. Exercise reshapes the tumor microenvironment by regulating cytokines (e.g., IL-10, TGF-β, IFN-γ), enhancing antitumor responses. It complements atezolizumab (a PD-L1 inhibitor) by counteracting PD-1/PD-L1 immune evasion. **(D)** Prospects and challenges. In nasopharyngeal cancer, exercise reduces IL-6, TNF-α, and CRP while activating NK cells, CTLs, and macrophages. Integrating exercise with drug therapy holds promise for improving therapeutic efficacy.

Allergic rhinitis (AR), affecting up to 40% globally, is an IgE-mediated nasal inflammation often comorbid with asthma, triggered by seasonal or perennial allergens (38, 39). Pharmacotherapy efficacy is limited, prompting interest in immune-regulating strategies (40). Moderate exercise enhances T, B, and NK cell activity, suppresses pro-inflammatory cytokines, and strengthens systemic/local immunity (**Figure 1**) (41). In the nasal cavity, it improves blood flow, immune cell aggregation, and microvascular permeability, optimizing drug delivery (42). At the immune signaling level, exercise promotes T cell migration, activation, and IL-10/TGF-β release within local mucosal niches, thereby reshaping inflammatory pathways and reducing hypersensitivity (43, 44). It also mitigates allergic sensitization, though excessive exercise may worsen airway irritation (45). Individualized intensity programs are thus essential.

### 2.2 Exercise enhances pharmacological efficacy in rhinitis

Exercise improves immune regulation in AR and enhances drug efficacy by boosting circulation and tissue drug delivery (11, 46), allowing lower doses and fewer side effects. Montelukast (MT), a leukotriene D4 receptor antagonist for asthma, AR, and EIB, shows variable efficacy (47, 48). Exercise may enhance MT pharmacokinetics/dynamics through improved hemodynamics and modulation of immune-related signaling pathways regulating drug transporters and metabolism (**Figure 1**) (49). Increased anti-inflammatory cytokines can further potentiate MT’s effects, reducing dose needs (50). Pulmonary benefits, including improved ventilation, enhanced mucociliary clearance, and greater elasticity, also contribute to better responsiveness (51, 52). Thus, MT plus moderate exercise integrates drug action with immune signal regulation, making it a promising AR strategy warranting optimization studies.

### 2.3 Immune evasion mechanisms in nasopharyngeal carcinoma tumor microenvironment

Nasopharyngeal carcinoma (NPC) exhibits strong immune evasion within the tumor microenvironment (TME), driven by tumor–immune–stroma interactions (53). Key mechanisms include upregulation of immune checkpoints such as PD-1/PD-L1 and CTLA-4, with PD-1 binding to PD-L1 inducing T cell exhaustion and weakening antitumor immunity. NPC also secretes TGF-β and IL-10, promoting regulatory T cell (Treg) and myeloid-derived suppressor cell (MDSC) infiltration, further suppressing immunity (54). Additionally, tumor-associated fibroblasts and endothelial cells release matrix metalloproteinases (MMPs) and remodel the extracellular matrix, hindering immune cell infiltration and reinforcing an immunosuppressive niche. These mechanisms reshape immune signal transduction networks within the TME, limiting effector cell function and promoting tumor persistence.

### 2.4 Exercise and immune checkpoint inhibitors in nasopharyngeal carcinoma

Immune checkpoint inhibitors (ICIs), including anti-PD-1/PD-L1 and anti-CTLA-4, improve NPC treatment by reactivating CTLs and NK cells, though efficacy is limited by the immunosuppressive TME (55). Exercise enhances ICI effects through modulation of immune signaling, boosting T cell function, increasing CD8^+^ T and NK cell infiltration into the TME, reducing PD-1/CTLA-4 expression, alleviating hypoxia, and improving blood flow, thereby promoting antigen recognition (35). Clinical studies show exercise during ICI therapy improves OS, PFS, and immune profiles, while reducing fatigue and adverse events (56). Preclinical data suggest exercise delays NPC growth and shifts the TME toward immunostimulation, overcoming resistance. Combining exercise with ICIs refines immune signal transduction within the TME, offering a promising strategy to enhance efficacy and patient outcomes (57).

### 2.5 Exercise, tumor oxygenation, and immunotherapy enhancement in nasopharyngeal carcinoma

In NPC, tumor hypoxia drives immune evasion, therapy resistance, and aggressiveness (58). Aerobic/endurance exercise alleviates hypoxia by enhancing cardiovascular output, angiogenesis, and vascular normalization (59), reducing interstitial fluid pressure and improving oxygen diffusion, thereby lowering HIF-1α activity (60). This reversal decreases immunosuppressive myeloid-derived suppressor cells and Tregs, reprograms macrophages toward anti-tumor activity, and boosts CD8^+^ T cell infiltration and survival. At the single-cell level, improved oxygenation synergizes with PD-1/PD-L1 inhibitors, cancer vaccines, and adoptive T cell transfer by enhancing antigen presentation, limiting T cell exhaustion, and increasing effector cytokine production (61). Preclinical NPC studies support structured aerobic exercise as a non-pharmacological adjuvant to immunotherapy.

### 2.6 Exercise as an adjunct in nasopharyngeal carcinoma immunotherapy

Immunotherapy, particularly ICIs such as anti-PD-1/PD-L1 agents, has transformed treatment landscapes in multiple cancers, including NPC (62, 63). However, response rates to ICIs in NPC remain modest due to immune escape and an immunosuppressive TME (64). This immunosuppressive TME is shaped by dynamic interactions among tumor-associated immune cells, which can now be characterized at single-cell resolution to dissect resistance-related signal transduction. Exercise increases CD8^+^ T cell infiltration, reduces immunosuppressive factors like TGF-β and IL-10, and improves oxygenation in hypoxic tumor regions, thereby amplifying cytotoxic immune responses (**Figure 1**) (65). It also fine-tunes inflammatory and immune signaling pathways, promoting anti-tumor cytokine secretion and systemic immune activation (66). In NPC specifically, combining Atezolizumab with exercise may improve outcomes by reversing tumor immune evasion. This could involve enhanced antigen presentation and improved spatial organization of immune infiltrates, both of which are crucial for immunotherapy efficacy. Moreover, exercise could potentiate the effects of nano-platform photodynamic therapies that induce immunogenic cell death, further enhancing treatment synergy (67).

### 2.7 Clinical trials and real-world evidence

Clinical studies, including randomized trials and real-world evidence, show that exercise improves immune regulation in AR and NPC, enhancing therapeutic outcomes and quality of life. In AR, RCTs have demonstrated that moderate-intensity exercise reduces airway inflammation, relieves nasal congestion, and boosts the effects of antihistamines and nasal corticosteroids by increasing anti-inflammatory cytokines (IL-10) and decreasing pro-inflammatory cytokines (TNF-α). Real-world studies also suggest that higher physical activity levels correlate with fewer rhinitis exacerbations and less need for medical interventions. In NPC, clinical trials indicate that structured exercise combined with immunotherapy enhances immune responses, with increased T-cell infiltration and reduced PD-1/PD-L1 expression, suggesting synergy between exercise and immunotherapy. Cohort studies also highlight that single-cell immune profiling reveals exercise-induced shifts in TME composition, linking clinical benefit to rewired immune signal transduction (68, 69).

### 2.8 Clinical translation and emerging combinatorial strategies

Although Atezolizumab monotherapy has shown limited efficacy in NPC (ORR ~5%) in Chinese cohorts, combining it with chemotherapy improves outcomes (70). Exercise may complement such combinatorial strategies by improving drug delivery through enhanced perfusion and reprogramming immune cell signal transduction in the tumor milieu (71). These effects are increasingly appreciated through spatial transcriptomics and multi-omics analyses, which reveal how localized perfusion and immune cell positioning impact drug response. In addition to ICIs, new therapeutic agents such as Camrelizumab and the natural compound cinobufagin have shown promise in NPC. Cinobufagin induces cell cycle arrest and apoptosis in NPC cells and may be a candidate for integration into multimodal treatment strategies (72). Exercise could potentially enhance these agents’ efficacy by improving immune surveillance and systemic metabolism (73). Combining exercise with immune-activating compounds may remodel the tumor immune landscape, making it more responsive to targeted agents. Together, these findings point toward a multimodal, patient-specific approach that integrates ICIs, novel agents, and exercise-based interventions to improve NPC outcomes (74).

### 2.9 Challenges and future perspectives in exercise-drug integration

While the immunomodulatory potential of exercise is clear, its integration with pharmacological therapies presents several challenges. Exercise has a dual role: it can reduce tumor-promoting inflammation and enhance immune activation, but excessive intensity or duration may lead to immune suppression and treatment resistance (75). The key lies in tailoring exercise regimens to individual patient profiles, including cancer type, treatment phase, and physical condition (76). Interindividual variability in treatment response, tolerance to physical activity, and underlying comorbidities further complicate standardization (77). For example, frail patients may experience adverse effects from even mild exertion, while fitter patients may require higher intensity to achieve benefits. Personalized prescriptions, ideally integrated into routine care and synchronized with treatment windows, may optimize outcomes (78). Patient compliance also remains a hurdle. Fatigue, psychological burden, and side effects of treatment often limit adherence to structured exercise programs (79). Incorporating behavioral support, supervised group programs, and flexibility in exercise formats could help sustain participation and improve treatment adherence (80). Future research should leverage single-cell and systems-level analyses to refine exercise–drug integration strategies, ensuring alignment with immune signal transduction mechanisms in the TME.

## 3 Preclinical and clinical evidence on exercise-drug synergy in rhinitis and nasopharyngeal carcinoma

### 3.1 Animal studies on exercise-enhanced drug efficacy

**Figure 2:**
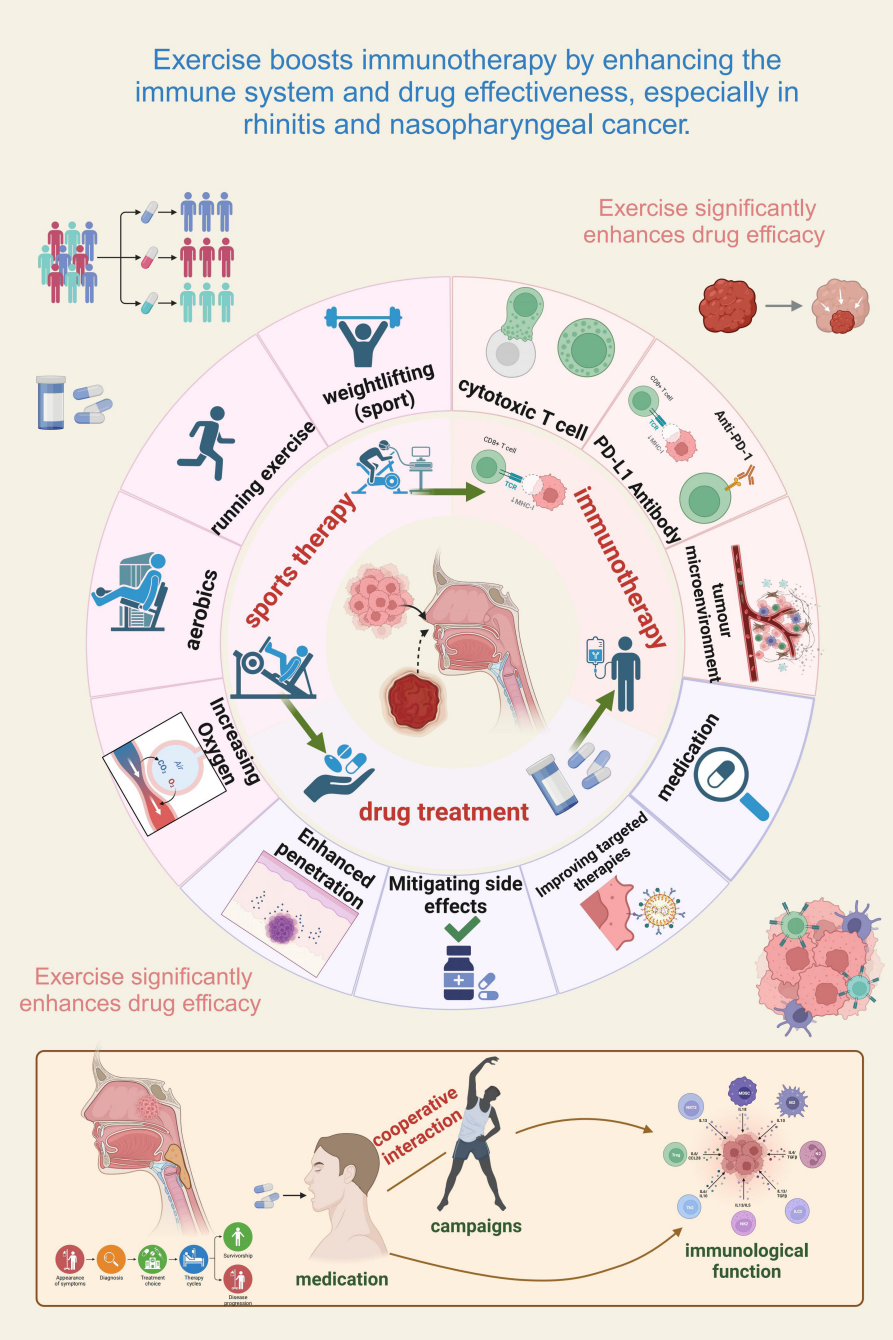
Exercise enhances immunotherapy efficacy by modulating immunity and drug effectiveness in rhinitis and nasopharyngeal cancer. Exercise strengthens immune function and improves drug treatment outcomes in immune-related conditions. The figure highlights the interplay of physical activity, immune modulation, and drug therapy in optimizing efficacy. Different exercise forms (e.g., running, weightlifting, aerobics) enhance oxygenation, drug delivery, and cytotoxic T cell activity. Exercise also synergizes with immunotherapy by fostering anti-tumor immunity, fortifying the tumor microenvironment, and boosting PD-L1 antibody effectiveness. In addition, it mitigates side effects, facilitates targeted therapy, and increases immune checkpoint inhibitor efficiency. The lower panel illustrates the cooperative interaction of exercise and medication, underscoring physical activity as a valuable adjunct to conventional treatment.

Preclinical studies highlight the potential of combining exercise with pharmacological interventions to enhance immune regulation in AR and NPC. In AR models, exercise activates immune cells (CD4^+^, CD8^+^ T cells, dendritic cells) and shifts the immune response toward Th1 dominance, reducing Th2-mediated allergic reactions (**Figure 2**) (26, 81).

Exercise also promotes anti-inflammatory cytokines like IL-10 and TGF-β, amplifying drug efficacy (82). In NPC models, exercise reprograms the tumor microenvironment (TME) by modulating inflammatory signaling and immune infiltration, reducing immunosuppressive mechanisms and boosting anti-tumor immune cell activity (83). Single-cell RNA sequencing (scRNA-seq) provides insights into the molecular effects of exercise, revealing how exercise reshapes immune signal transduction in cell subsets in AR (e.g., Tregs, Th2 cells) and NPC (e.g., cytotoxic CD8^+^ T cells, TAM reprogramming) (84–86).

Exercise also enhances immune checkpoint inhibitor efficacy by reducing MDSCs and Tregs, improving immune balance and therapeutic response (**Figure 2**) (81). Aerobic exercise reduces chronic inflammation, improving immunotherapy outcomes (66) and induces epigenetic and transcriptional changes within immune pathways, sensitizing tumors to treatments (87). Imaging studies show exercise improves tumor perfusion and vessel normalization, enhancing drug delivery.

### 3.2 Clinical insights into exercise and immunotherapy

Preliminary clinical studies suggest that exercise is a beneficial adjuvant in cancer immunotherapy, especially with checkpoint inhibitors (e.g., anti-PD-1/PD-L1) (56). For instance, moderate-intensity aerobic exercise alongside checkpoint inhibition in non-small cell lung cancer patients boosts immune surveillance, enhances CD8^+^ T cell activation, and reduces immunosuppressive cell populations such as Tregs and MDSCs (88). Similar trends in NPC trials show that exercise enhances NK and dendritic cell function, improves drug delivery, and reduces immune suppression (89). Exercise likely modulates TME intercellular signaling and chemokine-mediated communication (e.g., CXCL9/10), thereby regulating immune cell infiltration (90). In allergic rhinitis, exercise restores immune balance disrupted by chronic allergens, enhancing immune responses, reducing inflammation, and improving drug efficacy in symptom management (**Figure 2**) (91). Advances in single-cell profiling provide tools to map cell-type-specific immune signaling changes induced by combined exercise and immunotherapy (92). Despite promising results, challenges remain in defining optimal exercise regimens (type, intensity, duration, timing) for individual patients. The ACSM guidelines recommend moderate-intensity aerobic exercise (60-70% HRmax, 30–60 min) as safe (93), with personalized prescriptions and real-time monitoring tools enhancing tailored interventions (94).

### 3.3 Clinical potential of exercise combined with immunotherapy in NPC

Emerging clinical evidence shows significant synergy between exercise and immunotherapies like Atezolizumab (anti-PD-L1 antibody) in NPC. Exercise enhances immune cell activation, promotes tumor infiltration, and reduces common side effects, such as fatigue, improving patient compliance and outcomes (95). Clinical studies also show that combined exercise interventions improve physical endurance, psychological well-being, and immune response (96). Exercise enhances tumor perfusion and oxygenation, increasing drug bioavailability and efficacy, addressing limitations of single-agent immunotherapy (65). Furthermore, exercise-induced changes in the tumor microenvironment (TME) may overcome primary and acquired resistance to immune checkpoint inhibitors. Additionally, exercise mitigates treatment-related side effects, supports overall health, and enables sustained drug administration. Clinical data also demonstrate exercise’s positive effects on emotional well-being and long-term treatment adherence, underscoring its broader clinical utility.

### 3.4 Personalized exercise interventions: tailoring approaches for clinical efficacy

Personalization of exercise interventions remains essential, considering variability in patient demographics, comorbidities, tumor biology, and treatment regimens. Comprehensive pre-exercise evaluations incorporating medical history, physical capability assessments, and disease staging are critical for effective customization of exercise prescriptions (97). For patients with cardiovascular or respiratory comorbidities, low-intensity aerobic activities such as walking or Tai Chi are recommended initially, with gradual intensity escalation (98). Patients with diabetes require close blood glucose monitoring combined with carefully timed aerobic and resistance exercises (99). Individuals experiencing musculoskeletal pain may benefit from low-impact exercises like aquatic therapy (100). These exercises can relieve pain while maintaining muscle strength and joint flexibility. Future strategies should also consider integrating exercise training with other supportive care modalities, such as nutritional interventions or psychosocial counseling, to improve adherence and holistic patient outcomes. Importantly, integrating personalized exercise with immune monitoring at the single-cell level may optimize safety and efficacy (37).

### 3.5 Future directions and challenges

Future research should focus on the molecular and cellular mechanisms by which exercise regulates immune function in cancer and allergic diseases. Investigating how exercise affects immune cell subsets, cytokine profiles, and metabolic pathways will help optimize exercise regimens to enhance drug efficacy and reduce side effects (19).

Identifying and validating biomarkers to predict treatment response is essential. Integrating genomics, transcriptomics, proteomics, and advanced single-cell and spatial approaches (e.g., spatial CITE-seq, CRISPR screening) offers powerful strategies to uncover immune signaling mechanisms and design personalized interventions (101). However, challenges remain, including individual variability in response to exercise. Personalized interventions are needed to prevent immune overstimulation or excessive exercise-induced stress (102). Balancing exercise intensity to optimize immune regulation without exacerbating side effects or immune-related adverse events is key (103). Systematic research and well-designed clinical trials are required to define optimal parameters for combining exercise with drug therapies, ensuring safe and effective clinical translation (104).

## 4 Conclusion

The interplay between exercise and immune signaling is a promising, yet underexplored, pathway for enhancing the effectiveness of immunotherapy, particularly in conditions such as allergic rhinitis (AR) and nasopharyngeal carcinoma (NPC). Current evidence suggests that exercise influences immune cell activation, remodels inflammatory signaling within the tumor microenvironment (TME), and improves drug pharmacokinetics, collectively contributing to better therapeutic outcomes. Recent advances in single-cell technologies have highlighted the substantial heterogeneity in immune cell states and immune signal transduction pathways within the TME. Exercise-induced changes in immune cell distribution and function likely reshape these dynamic signaling processes at single-cell resolution. However, the precise molecular mechanisms by which exercise influences immune signaling and intercellular communication remain largely unknown. To address this, future research should integrate exercise immunology with single-cell transcriptomics and spatial omics technologies. These approaches will provide high-resolution insights into how exercise modulates immune cell populations and their interactions within disease-relevant tissues. Such integration could uncover new regulatory pathways and therapeutic targets, facilitating the development of personalized interventions that combine physical activity with immune-based treatments. By bridging exercise science with cutting-edge immune profiling technologies, researchers can uncover novel mechanisms supporting exercise as an adjunct to immunotherapy. This interdisciplinary strategy, focused on inflammation and immune signal transduction in the TME, may ultimately lead to more effective, individualized treatments for cancer and other immune-mediated diseases.

## References

[1] Nystoriak MA Bhatnagar A. Cardiovascular effects and benefits of exercise. Front Cardiovasc Med. (2018) 5:135. doi: 10.3389/fcvm.2018.00135, PMID: 30324108 PMC6172294

[2] Hellsten Y Nyberg M. Cardiovascular adaptations to exercise training. In: Prakash YS, editor. Comprehensive Physiology. Wiley, Hoboken, NJ, USA (John Wiley & Sons, Inc.) (2015). p. 1–32. doi: 10.1002/cphy.c140080, PMID: 26756625

[3] Yadav VD Salunkhe DS Lokhande VY. Multiparticulate drug delivery of losartan potassium via extrusion-spheronization: formulation and dissolution comparisons. BIOI. (2024) 5:1–11. doi: 10.15212/bioi-2024-0079

[4] Małkiewicz MA Szarmach A Sabisz A Cubała WJ Szurowska E Winklewski PJ. Blood-brain barrier permeability and physical exercise. J Neuroinflamm. (2019) 16:15. doi: 10.1186/s12974-019-1403-x, PMID: 30678702 PMC6345022

[5] Vaccarezza M. Physical exercise as chemosensitizer. Clin Exp Med. (2015) 15:427–7. doi: 10.1007/s10238-014-0312-7, PMID: 25200036

[6] Ohki M Hasegawa M Kurita N Watanabe I. Effects of exercise on nasal resistance and nasal blood flow. Acta Oto-Laryngologica. (1987) 104:328–33. doi: 10.3109/00016488709107336, PMID: 3673563

[7] Lenz TL. The effects of high physical activity on pharmacokinetic drug interactions. Expert Opin Drug Metab Toxicol. (2011) 7:257–66. doi: 10.1517/17425255.2011.553190, PMID: 21244343

[8] Ylitalo P. Effect of exercise on pharmacokinetics. Ann Med. (1991) 23:289–94. doi: 10.3109/07853899109148062, PMID: 1930919

[9] Sastre J. Ebastine in the treatment of allergic rhinitis and urticaria: 30 years of clinical studies and real-world experience. J Investig Allergol Clin Immunol. (2020) 30:156–68. doi: 10.18176/jiaci.0401, PMID: 30977465

[10] Frare RG Singh AK. A critical review of physicochemical properties and analytical methods applied to quantitative determination of ebastine. Crit Rev Analytical Chem. (2018) 48:102–9. doi: 10.1080/10408347.2017.1412816, PMID: 29235880

[11] Simpson RJ Kunz H Agha N Graff R. Exercise and the regulation of immune functions. In: Progress in Molecular Biology and Translational Science. Cambridge, MA, USA: Elsevier (2015). p. 355–80. doi: 10.1016/bs.pmbts.2015.08.001, PMID: 26477922

[12] Yang JH Bhargava P McCloskey D Mao N Palsson BO Collins JJ. Antibiotic-induced changes to the host metabolic environment inhibit drug efficacy and alter immune function. Cell Host Microbe. (2017) 22:757–765.e3. doi: 10.1016/j.chom.2017.10.020, PMID: 29199098 PMC5730482

[13] Wilkinson EM Ilhan ZE Herbst-Kralovetz MM. Microbiota–drug interactions: Impact on metabolism and efficacy of therapeutics. Maturitas. (2018) 112:53–63. doi: 10.1016/j.maturitas.2018.03.012, PMID: 29704918

[14] Islam N Irfan M Zahoor AF Iqbal MS Syed HK Khan IU. Improved bioavailability of ebastine through development of transfersomal oral films. Pharmaceutics. (2021) 13:1315. doi: 10.3390/pharmaceutics13081315, PMID: 34452276 PMC8401636

[15] Islam N Irfan M Khan S-U-D Syed HK Iqbal MS Khan IU. Poloxamer-188 and d-α-tocopheryl polyethylene glycol succinate (TPGS-1000) mixed micelles integrated orodispersible sublingual films to improve oral bioavailability of ebastine; *in vitro* and *in vivo* characterization. Pharmaceutics. (2021) 13:54. doi: 10.3390/pharmaceutics13010054, PMID: 33406587 PMC7823785

[16] Le T Aguilar B Mangal JL Acharya AP. Oral drug delivery for immunoengineering. Bioengineering Transla Med. (2022) 7:e10243. doi: 10.1002/btm2.10243, PMID: 35111945 PMC8780903

[17] Wang J Liu S Li G Xiao J. Exercise regulates the immune system. In: Xiao J, editor. Physical Exercise for Human Health. Advances in Experimental Medicine and Biology. Springer Nature Singapore, Singapore (2020). p. 395–408. doi: 10.1007/978-981-15-1792-1_27, PMID: 32342473

[18] McCullough DJ Stabley JN Siemann DW Behnke BJ. Modulation of blood flow, hypoxia, and vascular function in orthotopic prostate tumors during exercise. JNCI. (2014) 106:dju036. doi: 10.1093/jnci/dju036, PMID: 24627275 PMC3982888

[19] Gustafson MP Wheatley-Guy CM Rosenthal AC Gastineau DA Katsanis E Johnson BD. Exercise and the immune system: taking steps to improve responses to cancer immunotherapy. J Immunother Cancer. (2021) 9:e001872. doi: 10.1136/jitc-2020-001872, PMID: 34215686 PMC8256759

[20] Ebadi P Karimi MH Amirghofran Z. Plant components for immune modulation targeting dendritic cells: implication for therapy. Immunotherapy. (2014) 6:1037–53. doi: 10.2217/imt.14.77, PMID: 25428644

[21] Yao J Shen Q Huang M Ding M Guo Y Chen W. Screening tumor specificity targeted by arnicolide D, the active compound of Centipeda minima and molecular mechanism underlying by integrative pharmacology. J Ethnopharmacology. (2022) 282:114583. doi: 10.1016/j.jep.2021.114583, PMID: 34487850

[22] Gondhowiardjo SA Adham M Lisnawati Kodrat H Tobing DL Handoko. Current immune-related molecular approach in combating nasopharyngeal cancer. World J Oncol. (2019) 10:157–61. doi: 10.14740/wjon1214, PMID: 31636788 PMC6785271

[23] Allen J Sun Y Woods JA. Exercise and the regulation of inflammatory responses. In: Progress in Molecular Biology and Translational Science. Cambridge, MA, USA: Elsevier (2015). p. 337–54. doi: 10.1016/bs.pmbts.2015.07.003, PMID: 26477921

[24] Idorn M Hojman P. Exercise-dependent regulation of NK cells in cancer protection. Trends Mol Med. (2016) 22:565–77. doi: 10.1016/j.molmed.2016.05.007, PMID: 27262760

[25] Cavkaytar O Akdis CA Akdis M. Modulation of immune responses by immunotherapy in allergic diseases. Curr Opin Pharmacol. (2014) 17:30–7. doi: 10.1016/j.coph.2014.07.003, PMID: 25062122

[26] Idorn M Thor Straten P. Exercise and cancer: from “healthy” to “therapeutic”? Cancer Immunol Immunother. (2017) 66:667–71. doi: 10.1007/s00262-017-1985-z, PMID: 28324125 PMC5406418

[27] Fiuza-Luces C Valenzuela PL Castillo-García A Lucia A. Exercise benefits meet cancer immunosurveillance: implications for immunotherapy. Trends Cancer. (2021) 7:91–3. doi: 10.1016/j.trecan.2020.12.003, PMID: 33358110

[28] Ostrowski K Rohde T Asp S Schjerling P Pedersen BK. Pro- and anti-inflammatory cytokine balance in strenuous exercise in humans. J Physiol. (1999) 515:287–91. doi: 10.1111/j.1469-7793.1999.287ad.x, PMID: 9925898 PMC2269132

[29] Martin SA Pence BD Woods JA. Exercise and respiratory tract viral infections. Exercise Sport Sci Rev. (2009) 37:157–64. doi: 10.1097/JES.0b013e3181b7b57b, PMID: 19955864 PMC2803113

[30] Mustian KM Sprod LK Janelsins M Peppone LJ Mohile S. Exercise recommendations for cancer-related fatigue, cognitive impairment, sleep problems, depression, pain, anxiety, and physical dysfunction—A review. Oncol Hematol Rev (US). (2012) 08:81. doi: 10.17925/OHR.2012.08.2.81, PMID: 23667857 PMC3647480

[31] Buss LA Williams T Hock B Ang AD Robinson BA Currie MJ. Effects of exercise and anti-PD-1 on the tumour microenvironment. Immunol Lett. (2021) 239:60–71. doi: 10.1016/j.imlet.2021.08.005, PMID: 34480981

[32] Campbell JP Turner JE. Debunking the myth of exercise-induced immune suppression: redefining the impact of exercise on immunological health across the lifespan. Front Immunol. (2018) 9:648. doi: 10.3389/fimmu.2018.00648, PMID: 29713319 PMC5911985

[33] Nieman DC. Marathon training and immune function. Sports Med. (2007) 37:412–5. doi: 10.2165/00007256-200737040-00036, PMID: 17465622

[34] Guo Z Gao J Liu L Liu X. Quantitatively predicting effects of exercise on pharmacokinetics of drugs using a physiologically based pharmacokinetic model. Drug Metab Disposition. (2024) 52:1271–87. doi: 10.1124/dmd.124.001809, PMID: 39251368

[35] Gomes-Santos IL Amoozgar Z Kumar AS Ho WW Roh K Talele NP. Exercise training improves tumor control by increasing CD8+ T-cell infiltration via CXCR3 signaling and sensitizes breast cancer to immune checkpoint blockade. Cancer Immunol Res. (2021) 9:765–78. doi: 10.1158/2326-6066.CIR-20-0499, PMID: 33839688 PMC8295193

[36] Esteves M Monteiro MP Duarte JA. Role of regular physical exercise in tumor vasculature: favorable modulator of tumor milieu. Int J Sports Med. (2021) 42:389–406. doi: 10.1055/a-1308-3476, PMID: 33307553

[37] Plaza-Florido A Lucia A Radom-Aizik S Fiuza-Luces C. Anticancer effects of exercise: Insights from single-cell analysis. J Sport Health Sci. (2024) 13:676–8. doi: 10.1016/j.jshs.2024.01.008, PMID: 38266673 PMC11282339

[38] Licari A Magri P De Silvestri A Giannetti A Indolfi C Mori F. Epidemiology of allergic rhinitis in children: A systematic review and meta-analysis. J Allergy Clin Immunol. (2023) 11:2547–56. doi: 10.1016/j.jaip.2023.05.016, PMID: 37236349

[39] Nur Husna SM Tan H-TT Md Shukri N Mohd Ashari NS Wong KK. Allergic rhinitis: A clinical and pathophysiological overview. Front Med. (2022) 9:874114. doi: 10.3389/fmed.2022.874114, PMID: 35463011 PMC9021509

[40] Campuzano-Revilla GP. Allergic Rhinitis: keys for the clinician. Mex J Med Res. (2022) 10:34–40. doi: 10.29057/mjmr.v10i19.7739

[41] Kristian H Pelealu OCP Mengko SK. Efek olahraga terhadap perbaikan gejala rinitis alergi. JBM. (2022) 14:46. doi: 10.35790/jbm.v14i1.37562

[42] Côté A Turmel J Boulet L-P. Exercise and asthma. Semin Respir Crit Care Med. (2018) 39:019–28. doi: 10.1055/s-0037-1606215, PMID: 29427982

[43] Fernandes P De Mendonça Oliveira L Brüggemann TR Sato MN Olivo CR Arantes-Costa FM. Physical exercise induces immunoregulation of TREG, M2, and pDCs in a lung allergic inflammation model. Front Immunol. (2019) 10:854. doi: 10.3389/fimmu.2019.00854, PMID: 31156611 PMC6532549

[44] Lambrecht BN Hammad H. The immunology of asthma. Nat Immunol. (2015) 16:45–56. doi: 10.1038/ni.3049, PMID: 25521684

[45] Park J Park JH Park J Choi J Kim TH. Association between allergic rhinitis and regular physical activity in adults: A nationwide cross-sectional study. IJERPH. (2020) 17:5662. doi: 10.3390/ijerph17165662, PMID: 32764473 PMC7459676

[46] Simpson RJ Boßlau TK Weyh C Niemiro GM Batatinha H Smith KA. Exercise and adrenergic regulation of immunity. Brain Behavior Immun. (2021) 97:303–18. doi: 10.1016/j.bbi.2021.07.010, PMID: 34302965

[47] Paggiaro P Bacci E. Montelukast in asthma: a review of its efficacy and place in therapy. Ther Adv Chronic Dis. (2011) 2:47–58. doi: 10.1177/2040622310383343, PMID: 23251741 PMC3513871

[48] Nayak A. A review of montelukast in the treatment of asthma and allergic rhinitis. Expert Opin Pharmacotherapy. (2004) 5:679–86. doi: 10.1517/14656566.5.3.679, PMID: 15013935

[49] Somani SM Gupta SK Frank S Corder CN. Effect of exercise on disposition and pharmacokinetics of drugs. Drug Dev Res. (1990) 20:251–75. doi: 10.1002/ddr.430200302

[50] Bonsignore MR La Grutta S Cibella F Scichilone N Cuttitta G Interrante A. Effects of exercise training and montelukast in children with mild asthma. Med Sci Sports Exercise. (2008) 40:405–12. doi: 10.1249/MSS.0b013e31815d9670, PMID: 18379200

[51] Baldwin DR Hill AL Peckham DG Knox AJ. Effect of addition of exercise to chest physiotherapy on sputum expectoration and lung function in adults with cystic fibrosis. Respir Med. (1994) 88:49–53. doi: 10.1016/0954-6111(94)90174-0, PMID: 8029514

[52] Ni R Cai L Xing Y Fan X. The effects of respiratory training combined with limb exercise on pulmonary function and quality of life in patients with bronchiectasis. JMDH. (2023) 16:475–82. doi: 10.2147/JMDH.S388944, PMID: 36861133 PMC9968867

[53] Su ZY Siak PY Leong C-O Cheah S-C. Nasopharyngeal carcinoma and its microenvironment: past, current, and future perspectives. Front Oncol. (2022) 12:840467. doi: 10.3389/fonc.2022.840467, PMID: 35311066 PMC8924466

[54] Gong L Kwong DL-W Dai W Wu P Wang Y Lee AW-M. The stromal and immune landscape of nasopharyngeal carcinoma and its implications for precision medicine targeting the tumor microenvironment. Front Oncol. (2021) 11:744889. doi: 10.3389/fonc.2021.744889, PMID: 34568077 PMC8462296

[55] Wang S Chen S Zhong Q Liu Y. Immunotherapy for the treatment of advanced nasopharyngeal carcinoma: a promising new era. J Cancer Res Clin Oncol. (2023) 149:2071–9. doi: 10.1007/s00432-022-04214-8, PMID: 35876949 PMC11798144

[56] Verheijden RJ Cabané Ballester A Smit KC Van Eijs MJM Bruijnen CP Van Lindert ASR. Physical activity and checkpoint inhibition: association with toxicity and survival. JNCI. (2024) 116:573–9. doi: 10.1093/jnci/djad245, PMID: 38001030 PMC10995850

[57] Brummer C Pukrop T Wiskemann J Bruss C Ugele I Renner K. Can exercise enhance the efficacy of checkpoint inhibition by modulating anti-tumor immunity? Cancers. (2023) 15:4668. doi: 10.3390/cancers15184668, PMID: 37760634 PMC10526963

[58] Liu H Tang L Li Y Xie W Zhang L Tang H. Nasopharyngeal carcinoma: current views on the tumor microenvironment’s impact on drug resistance and clinical outcomes. Mol Cancer. (2024) 23:20. doi: 10.1186/s12943-023-01928-2, PMID: 38254110 PMC10802008

[59] SChadler KL Thomas NJ Galie PA Bhang DH Roby KC Addai P. Tumor vessel normalization after aerobic exercise enhances chemotherapeutic efficacy. Oncotarget. (2016) 7:65429–40. doi: 10.18632/oncotarget.11748, PMID: 27589843 PMC5323166

[60] Jia N Zhou Y Dong X Ding M. The antitumor mechanisms of aerobic exercise: A review of recent preclinical studies. Cancer Med. (2021) 10:6365–73. doi: 10.1002/cam4.4169, PMID: 34387383 PMC8446393

[61] Hatfield SM Kjaergaard J Lukashev D Schreiber TH Belikoff B Abbott R. Immunological mechanisms of the antitumor effects of supplemental oxygenation. Sci Transl Med. (2015) 7:277ra30. doi: 10.1126/scitranslmed.aaa1260, PMID: 25739764 PMC4641038

[62] Yu Z. Immunotherapy breakthrough: immune checkpoint inhibitors in cancer treatment. HSET. (2024) 123:523–6. doi: 10.54097/eqy95j75

[63] Xu J-Y Wei X-L Wang Y-Q Wang F-H. Current status and advances of immunotherapy in nasopharyngeal carcinoma. Ther Adv Med Oncol. (2022) 14:17588359221096214. doi: 10.1177/17588359221096214, PMID: 35547095 PMC9083041

[64] Iyengar NM Jones LW. Development of exercise as interception therapy for cancer: A review. JAMA Oncol. (2019) 5:1620. doi: 10.1001/jamaoncol.2019.2585, PMID: 31436828 PMC7542631

[65] Wiggins JM Opoku-Acheampong AB Baumfalk DR Siemann DW Behnke BJ. Exercise and the tumor microenvironment: potential therapeutic implications. Exercise Sport Sci Rev. (2018) 46:56–64. doi: 10.1249/JES.0000000000000137, PMID: 29166299

[66] Spiliopoulou P Gavriatopoulou M Kastritis E Dimopoulos M Terzis G. Exercise-induced changes in tumor growth via tumor immunity. Sports. (2021) 9:46. doi: 10.3390/sports9040046, PMID: 33808154 PMC8065770

[67] Liu X Lu Y Li X Luo L You J. Nanoplatform-enhanced photodynamic therapy for the induction of immunogenic cell death. J Controlled Release. (2024) 365:1058–73. doi: 10.1016/j.jconrel.2023.11.058, PMID: 38056695

[68] Ren X Zhang L Zhang Y Li Z Siemers N Zhang Z. Insights gained from single-cell analysis of immune cells in the tumor microenvironment. Annu Rev Immunol. (2021) 39:583–609. doi: 10.1146/annurev-immunol-110519-071134, PMID: 33637019

[69] Zhu Z He M Zhang T Zhao T Qin S Gao M. LSD1 promotes the FSH responsive follicle formation by regulating autophagy and repressing Wt1 in the granulosa cells. Sci Bull. (2024) 69:1122–36. doi: 10.1016/j.scib.2024.01.015, PMID: 38302330

[70] Perri F. Locally advanced nasopharyngeal carcinoma: Current and emerging treatment strategies. WJCO. (2011) 2:377. doi: 10.5306/wjco.v2.i12.377, PMID: 22171280 PMC3235656

[71] Goliwas KF Deshane JS Elmets CA Athar M. Moving immune therapy forward targeting tme. Physiol Rev. (2021) 101:417–25. doi: 10.1152/physrev.00008.2020, PMID: 32790578 PMC8428923

[72] Dai C-L Zhang R An P Deng Y-Q Rahman K Zhang H. Cinobufagin: a promising therapeutic agent for cancer. J Pharm Pharmacol. (2023) 75:1141–53. doi: 10.1093/jpp/rgad059, PMID: 37390473

[73] Pan Z Luo Y Xia Y Zhang X Qin Y Liu W. Cinobufagin induces cell cycle arrest at the S phase and promotes apoptosis in nasopharyngeal carcinoma cells. Biomedicine Pharmacotherapy. (2020) 122:109763. doi: 10.1016/j.biopha.2019.109763, PMID: 31918288

[74] Oladejo M Tijani AO Puri A Chablani L. Adjuvants in cutaneous vaccination: A comprehensive analysis. J Controlled Release. (2024) 369:475–92. doi: 10.1016/j.jconrel.2024.03.045, PMID: 38569943

[75] Meneses-Echávez JF Correa-Bautista JE González-Jiménez E Schmidt Río-Valle J Elkins MR Lobelo F. The effect of exercise training on mediators of inflammation in breast cancer survivors: A systematic review with meta-analysis. Cancer Epidemiology Biomarkers Prev. (2016) 25:1009–17. doi: 10.1158/1055-9965.EPI-15-1061, PMID: 27197276

[76] Liu J Liu W Wan Y Mao W. Crosstalk between exercise and immunotherapy: current understanding and future directions. Research. (2024) 7:360. doi: 10.34133/research.0360, PMID: 38665847 PMC11045263

[77] Ramírez-Vélez R Izquierdo M. Editorial: precision physical activity and exercise prescriptions for disease prevention: the effect of interindividual variability under different training approaches. Front Physiol. (2019) 10:646. doi: 10.3389/fphys.2019.00646, PMID: 31178759 PMC6543979

[78] Buffart LM Sweegers MG May AM Chinapaw MJ Van Vulpen JK Newton RU. Targeting exercise interventions to patients with cancer in need: an individual patient data meta-analysis. JNCI. (2018) 110:1190–200. doi: 10.1093/jnci/djy161, PMID: 30299508 PMC6454466

[79] Cameron C. Patient compliance: recognition of factors involved and suggestions for promoting compliance with therapeutic regimens. J Advanced Nurs. (1996) 24:244–50. doi: 10.1046/j.1365-2648.1996.01993.x, PMID: 8858426

[80] Brinks J Franklin BA. Suboptimal exercise compliance: common barriers to an active lifestyle and counseling strategies to overcome them. Am J Lifestyle Med. (2011) 5:253–61. doi: 10.1177/1559827610391971

[81] Ashcraft KA Warner AB Jones LW Dewhirst MW. Exercise as adjunct therapy in cancer. Semin Radiat Oncol. (2019) 29:16–24. doi: 10.1016/j.semradonc.2018.10.001, PMID: 30573180 PMC6656408

[82] Dorneles GP Dos Passos AAZ Romão PRT Peres A. New insights about regulatory T cells distribution and function with exercise: the role of immunometabolism. CPD. (2020) 26:979–90. doi: 10.2174/1381612826666200305125210, PMID: 32133958

[83] Hojman P. Exercise protects from cancer through regulation of immune function and inflammation. Biochem Soc Trans. (2017) 45:905–11. doi: 10.1042/BST20160466, PMID: 28673937

[84] Cristallini C Rossin D Vanni R Barbani N Bulgheresi C Labardi M. A biodegradable, microstructured, electroconductive and nano-integrated drug eluting patch (MENDEP) for myocardial tissue engineering. Bioactive Materials. (2025) 50:246–72. doi: 10.1016/j.bioactmat.2025.04.008, PMID: 40270551 PMC12017858

[85] Hojman P Gehl J Christensen JF Pedersen BK. Molecular mechanisms linking exercise to cancer prevention and treatment. Cell Metab. (2018) 27:10–21. doi: 10.1016/j.cmet.2017.09.015, PMID: 29056514

[86] Luo S Wang L Xiao Y Cao C Liu Q Zhou Y. Single-cell RNA-sequencing integration analysis revealed immune cell heterogeneity in five human autoimmune diseases. BIOI. (2023) 4:145–59. doi: 10.15212/bioi-2023-0012

[87] Bei Y Wang H Liu Y Su Z Li X Zhu Y. Exercise-Induced miR-210 Promotes Cardiomyocyte Proliferation and Survival and Mediates Exercise-Induced Cardiac Protection against Ischemia/Reperfusion Injury. Research. (2024) 7:327. doi: 10.34133/research.0327, PMID: 38410280 PMC10895486

[88] Holmen Olofsson G Jensen AWP Idorn M Thor Straten P. Exercise oncology and immuno-oncology; A (Future) dynamic duo. IJMS. (2020) 21:3816. doi: 10.3390/ijms21113816, PMID: 32471301 PMC7312459

[89] Gielen S Hambrecht R. Treatment strategies in endothelial dysfunction: Physical exercise versus pharmacological therapy. Eur J Cardiovasc Prev Rehabil. (2005) 12:318–20. doi: 10.1097/01.hjr.0000174826.72022.c4, PMID: 16079637

[90] Miao S-N Chai M-Q Liu X-Y Wei C-Y Zhang C-C Sun N-N. Exercise accelerates recruitment of CD8+ T cell to promotes anti-tumor immunity in lung cancer via epinephrine. BMC Cancer. (2024) 24:474. doi: 10.1186/s12885-024-12224-7, PMID: 38622609 PMC11021002

[91] Casciano F Caruso L Zauli E Gonelli A Zauli G Vaccarezza M. Emerging mechanisms of physical exercise benefits in adjuvant and neoadjuvant cancer immunotherapy. Biomedicines. (2024) 12:2528. doi: 10.3390/biomedicines12112528, PMID: 39595094 PMC11591576

[92] Davis-Marcisak EF Deshpande A Stein-O’Brien GL Ho WJ Laheru D Jaffee EM. From bench to bedside: Single-cell analysis for cancer immunotherapy. Cancer Cell. (2021) 39:1062–80. doi: 10.1016/j.ccell.2021.07.004, PMID: 34329587 PMC8406623

[93] Buford TW Roberts MD Church TS. Toward exercise as personalized medicine. Sports Med. (2013) 43:157–65. doi: 10.1007/s40279-013-0018-0, PMID: 23382011 PMC3595541

[94] Wang Q Guo F Zhang Q Hu T Jin Y Yang Y. Organoids in gastrointestinal diseases: from bench to clinic. MedComm. (2024) 5:e574. doi: 10.1002/mco2.574, PMID: 38948115 PMC11214594

[95] Handford J Chen M Rai R Moss CL Enting D Peat N. Is there a role for exercise when treating patients with cancer with immune checkpoint inhibitors? A scoping review. Cancers. (2022) 14:5039. doi: 10.3390/cancers14205039, PMID: 36291823 PMC9599872

[96] Burnham TR Wilcox A. Effects of exercise on physiological and psychological variables in cancer survivors. Med Sci Sports Exercise. (2002) 34:1863–7. doi: 10.1097/00005768-200212000-00001, PMID: 12471288

[97] Xie W Lu D Liu S Li J Li R. The optimal exercise intervention for sleep quality in adults: A systematic review and network meta-analysis. Prev Med. (2024) 183:107955. doi: 10.1016/j.ypmed.2024.107955, PMID: 38641082

[98] Mittaz Hager A-G Mathieu N Lenoble-Hoskovec C Swanenburg J De Bie R Hilfiker R. Effects of three home-based exercise programmes regarding falls, quality of life and exercise-adherence in older adults at risk of falling: protocol for a randomized controlled trial. BMC Geriatr. (2019) 19:13. doi: 10.1186/s12877-018-1021-y, PMID: 30642252 PMC6332592

[99] Kirwan JP Sacks J Nieuwoudt S. The essential role of exercise in the management of type 2 diabetes. CCJM. (2017) 84:S15–21. doi: 10.3949/ccjm.84.s1.03, PMID: 28708479 PMC5846677

[100] Cento AS Leigheb M Caretti G Penna F. Exercise and exercise mimetics for the treatment of musculoskeletal disorders. Curr Osteoporos Rep. (2022) 20:249–59. doi: 10.1007/s11914-022-00739-6, PMID: 35881303 PMC9522759

[101] Holl EK Frazier VN Landa K Beasley GM Hwang ES Nair SK. Examining peripheral and tumor cellular immunome in patients with cancer. Front Immunol. (2019) 10:1767. doi: 10.3389/fimmu.2019.01767, PMID: 31417550 PMC6685102

[102] Hecksteden A Kraushaar J Scharhag-Rosenberger F Theisen D Senn S Meyer T. Individual response to exercise training - a statistical perspective. J Appl Physiol. (2015) 118:1450–9. doi: 10.1152/japplphysiol.00714.2014, PMID: 25663672

[103] Gleeson M. Immune function in sport and exercise. J Appl Physiol. (2007) 103:693–9. doi: 10.1152/japplphysiol.00008.2007, PMID: 17303714

[104] Ashcraft KA Peace RM Betof AS Dewhirst MW Jones LW. Efficacy and mechanisms of aerobic exercise on cancer initiation, progression, and metastasis: A critical systematic review of *in vivo* preclinical data. Cancer Res. (2016) 76:4032–50. doi: 10.1158/0008-5472.CAN-16-0887, PMID: 27381680 PMC5378389

